# Behçet’s Disease Mimicking Drug-Induced Mucocutaneous Lesions in a Patient on Anti-tuberculous Drugs

**DOI:** 10.7759/cureus.96922

**Published:** 2025-11-15

**Authors:** Maha Batool, Imman Akram, Farah Babar, Minahil Binte Irfan, Imran Khan

**Affiliations:** 1 Internal Medicine, Gulab Devi Teaching Hospital, Lahore, PAK; 2 Medicine, Gulab Devi Teaching Hospital, Lahore, PAK; 3 Internal Medicine, Gulab Devi Teaching Hospital/Al-Aleem Medical College, Lahore, PAK

**Keywords:** anti tubercular therapy (att), behçet’s disease, infectious disease triggers, ocular manifestations, pathergy test

## Abstract

Behçet’s disease is an inflammatory disease characterized by recurrent oral aphthous ulcers and numerous potential systemic manifestations. These include genital ulcers, skin lesions, and diseases affecting the eyes, nervous system, blood vessels, joints, and gastrointestinal tract. The underlying cause of the disease is unknown. As with other autoimmune diseases, the disorder may represent aberrant immune activity triggered by exposure to an agent, perhaps infectious, in patients with a genetic predisposition to develop the disease. We present the case of a 30-year-old male who developed features consistent with Behçet’s disease, including ocular, genital, and oral involvement, following treatment for presumed tuberculosis with anti-tuberculous therapy (ATT). As the oral aphthous ulcers are common in the general population and Behçet’s disease is relatively rare, the diagnosis should be considered in the context of recurrent aphthous ulcers accompanied by other systemic manifestations. There is no pathognomonic laboratory test for Behçet’s disease; therefore, the diagnosis is primarily clinical and meets the International Criteria for Behçet's Disease (ICBD) criteria. A score of 4 or more points is considered diagnostic. This publication underscores that Behçet’s disease is frequently misdiagnosed or mismanaged because of its overlapping clinical features with other disorders. Moreover, infectious triggers may, in some cases, contribute to the onset or unmasking of the disease.

## Introduction

Behçet disease, also known as the Silk Road disease, is a rare, chronic, autoimmune, multisystemic inflammatory disorder of unknown pathogenesis. The pooled prevalence of Behçet’s disease was calculated as 10.3 per 100,000 population globally, but it is particularly prevalent in the Middle East and Far East Asia. The presence of symptom clusters and regional differences in disease expression suggests the involvement of multiple pathological pathways involving genetic susceptibility, particularly HLA-B*51:01 [1-3]. Behçet’s disease mostly presents with mucocutaneous lesions, including recurrent oral and genital ulcers, uveitis, and skin lesions, as well as ocular manifestations and vasculitis [2]. Specifically, neutrophil hyperactivation and neutrophil-mediated tissue injury play a central role in Behçet’s syndrome by promoting endothelial dysfunction, platelet activation, and thrombogenesis [4]. Moreover, environmental triggers include microbial agents such as oral anaerobes [5], herpes simplex virus (HSV) [6], and Mycobacterium tuberculosis [7].

This case highlights the diagnostic challenges posed by overlapping symptoms between tuberculosis and Behçet’s disease. In countries with high tuberculosis prevalence, physicians have a high index of suspicion for it, leading to patients with Behçet disease initially receiving empirical anti-tuberculous therapy (ATT) before a definitive diagnosis is established, due to symptoms overlapping, such as fever, weight loss, ocular involvement, chronic oral lesions, or erythema nodosum-like lesions [7]. The coexistence or misdiagnosis of tuberculosis and Behçet’s disease poses significant diagnostic and therapeutic challenges, particularly given the scarcity of reported cases and retrospective studies from Pakistan. This report highlights the need to consider Behçet’s disease as a tuberculosis mimic with continuous clinical reassessment. The prevalence remains unknown, which further contributes to misdiagnosis [1].

However, the patient’s lack of improvement with antitubercular therapy, coupled with the subsequent appearance of hallmark manifestations of Behçet’s disease and a positive pathergy test, ultimately pointed toward an underlying autoimmune etiology rather than an infectious one.

## Case presentation

A 30-year-old male presented to the Medical Outpatient Department of Gulab Devi Teaching Hospital, Pakistan, in May 2025, with a complex multisystem presentation suggestive of Behçet’s disease. He reported recurrent bilateral ocular and visual disturbances that progressed to blurring of vision and inflammation, along with recurrent oral and genital ulcers for the past six months. The patient initially noticed blurring of vision during the eighth month of ATT, which he had been taking for a total duration of nine months, though treatment records were unavailable. He had returned from abroad two years earlier, after which he developed fever, shortness of breath, and weight loss. Based on these symptoms, he was empirically started on ATT in a peripheral healthcare facility for presumed tuberculosis and completed a full nine-month course of isoniazid, rifampicin, pyrazinamide, and ethambutol (HRZE) with good compliance about one year prior to presentation. By the eighth month of therapy, due to progressive visual blurring, he consulted an ophthalmologist and received treatment; however, his symptoms failed to improve.

**Figure 1:**
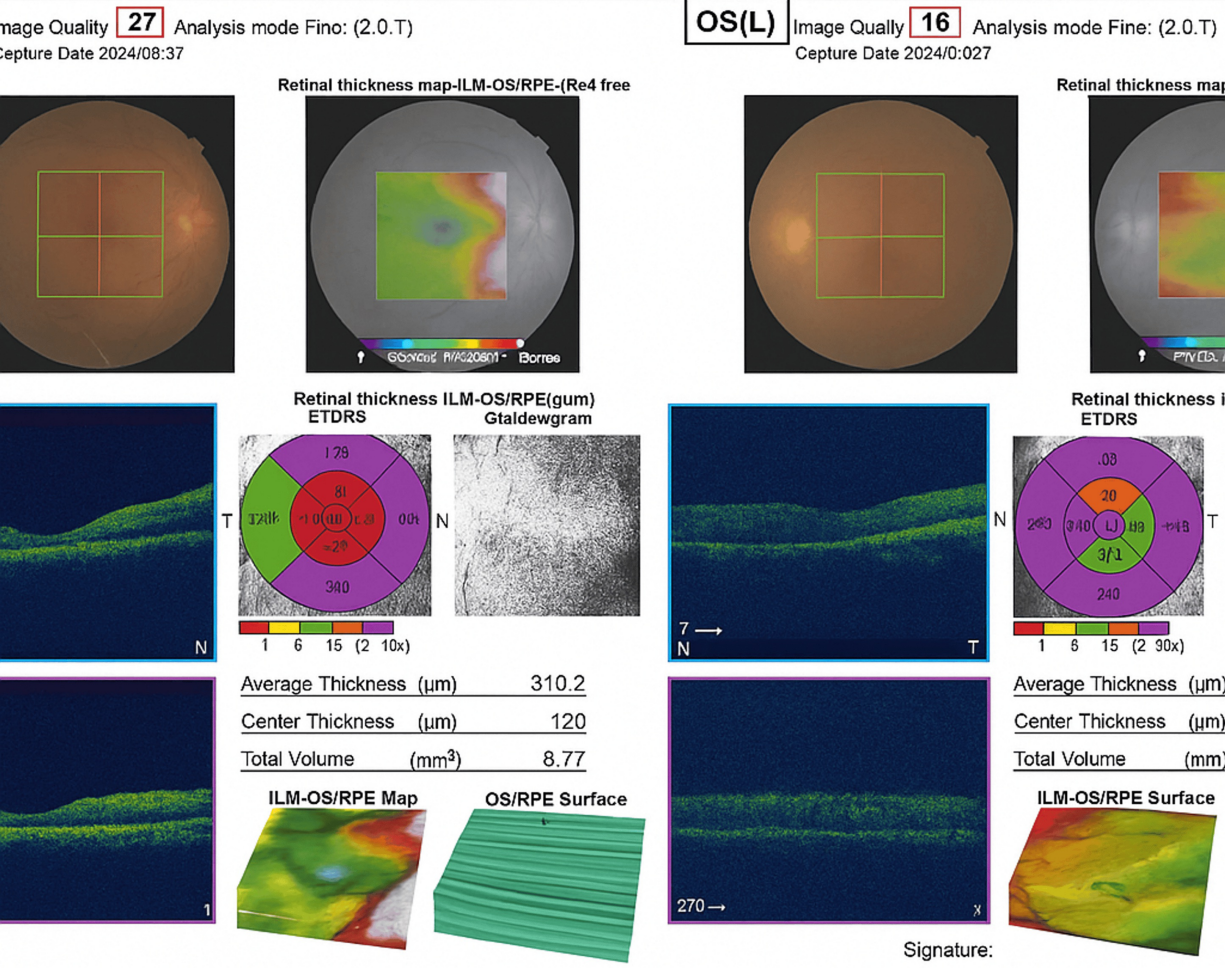
Optical coherence tomography (OCT) 3D Macula report from Topcon (Tokyo, Japan), showing macular scans of both eyes (OD: Right eye, OS: Left eye).

Ocular examination revealed markedly reduced visual acuity in both eyes, accompanied by refractive error (Table 1). Fundoscopic evaluation demonstrated characteristic findings consistent with ocular involvement in Behçet’s disease (Figure 1).

**Table 1:** Comprehensive ocular examination, including both anterior and posterior segments (fundoscopic) findings PR – Pupillary Reflex; PL – Perception of Light; CF – Counting Fingers; PH – Pinhole; N/I – No Improvement; EOM – Extraocular Muscle Movement; PSCC – Posterior Subcapsular Cataract.

Examination	Right eye	Left eye
Visual Acuity	CF (1m)	PL+, PR+
PH	N/I	N/I
EOM	Full	Full
Lids	Normal	Normal
Conjunctiva	Congestion	Congestion
Anterior Chamber	Faint flare	Marked flare
	+1 cells	+4 cells
Cornea	Pigmented KPs	Endothelial dusting
		1mm hypopyon
Pupil	Iris pigments on lens	Fibrinous exudates on pupillary margins
Lens	PSCC+	PSCC+
Vitreous	Vitreous haze grade III	Vitreous haze grade III
Retina	Peri-phlebitis	Peri-phlebitis
	Sheathing of vessels	Sheathing of vessels

In the right eye, pigmented keratic precipitates (KPs) with a faint flare were observed, along with endothelitis characterized by mutton-fat KPs and anterior chamber (AC) cells, consistent with anterior nongranulomatous uveitis. The optic disc appeared hyperemic, and there was Grade III vitreous haze with snowball opacities and multiple peripheral retinal inflammatory foci, which later progressed to Grade IV vitreous haze with numerous vitreous cells (Table 1).

In the left eye, corneal and aqueous congestion were evident, along with anterior chamber cells and flare, endothelitis with mutton-fat keratic precipitates, and anterior nongranulomatous uveitis localized to the pupillary margin. Similar to the right eye, optic disc hyperemia, Grade III vitreous haze, snowball opacities in the peripheral retina, and diffuse vitreous haze were also observed. Intraocular pressure was within normal limits in both eyes (Table 1).

Cross-sectional B-scans demonstrated retinal layer disruption, intraretinal inflammatory changes, possible cystoid macular changes, and surface irregularities consistent with inflammatory retinopathy. Optical coherence tomography (OCT) cross-sectional analysis in both eyes revealed disrupted retinal architecture, intraretinal fluid accumulation, and bilateral macular thickness variations indicative of inflammatory edema, along with irregularities of the retinal pigment (Figure 1).

The patient’s complete blood count (CBC) revealed normal hemoglobin and leukocyte levels; however, the mean corpuscular volume (MCV) was elevated (106 fL), indicating macrocytosis, which may be attributed to chronic illness or nutritional deficiency. The platelet count was within the lower-normal range, a finding that can also be observed in systemic inflammatory conditions (Table 2). The markedly elevated erythrocyte sedimentation rate (ESR) of 85 mm/hr (Table 2) strongly supports the presence of an ongoing systemic inflammatory process, consistent with the relapsing-remitting vasculitic nature of Behçet’s disease.

**Table 2:** Complete blood count, liver function test, and renal function test. CBC – Complete Blood Count; SGPT (ALT) – Serum Glutamic Pyruvic Transaminase / Alanine Aminotransferase; SGOT (AST) – Serum Glutamic Oxaloacetic Transaminase / Aspartate Aminotransferase; ALK – Alkaline Phosphatase; MCH – Mean Corpuscular Hemoglobin; MCV – Mean Corpuscular Volume; MCHC – Mean Corpuscular Hemoglobin Concentration; MPV – Mean Platelet Volume; HCT – Hematocrit; PCV – Packed Cell Volume

Investigation	Patient Value	Normal Range
Hemoglobin (g/dl)	13.2	13-18
Platelet Count (x10^3/uL)	342	150-450
White Blood Cell Count (x10^3/uL)	11.2	4-11
ESR (mm/hr)	85	1-10
HCT (PCV) (%)	40	40-54
MCV (fL)	106	76-96
MCH (pg)	31	27-32
MCHC (g/dl)	32	30-35
Neutrophils (%)	82	40-60
Lymphocytes (%)	14	20-40
Monocytes (%)	3	2-10
Eosinophils (%)	1	1-6
Bilirubin (mg/dl)	0.5	0.02-1.01
SGPT (IU/L)	27	5-42
SGOT (IU/L)	88	5-45
Alkaline Phosphatase (U/L)	304	80-306
Blood Urea (mg/dl)	20	10-50
Serum Creatinine (mg/dl)	0.8	0.6-1.1

The liver function tests showed elevated aspartate aminotransferase (AST) (88 U/L) and alkaline phosphatase (ALP) (304 U/L), which may reflect hepatic involvement secondary to systemic inflammation in Behçet’s disease or drug-induced hepatotoxicity related to prior ATT. Hepatotoxicity from ATT - particularly due to isoniazid, rifampicin, and pyrazinamide - is well documented, with transient elevations in aminotransferases occurring in up to 20% of patients and clinically significant hepatitis in approximately 2-5%. However, the absence of concurrent hyperbilirubinemia and normalization of other liver parameters in this patient favors an inflammatory or vasculitic etiology rather than ongoing drug-induced injury. Renal function tests were within normal limits (urea 20 mg/dL, creatinine 0.8 mg/dL), suggesting no evidence of renal involvement (Table 2).

Clinically, the correlation is strong. The patient presented with recurrent painful oral ulcerations located on the lateral borders of the tongue and the inner aspects of both cheeks, appearing as shallow, round lesions with bright red borders. Painful genital ulcers were also noted on the scrotum, characterized by shallow, round, erythematous lesions, which represent a hallmark diagnostic criterion. In addition, there was ocular involvement in the form of bilateral panuveitis and laboratory evidence of systemic inflammation. Collectively, these findings fulfill the major diagnostic criteria for Behçet’s disease and indicate multisystem vasculitic involvement.

**Figure 2:**
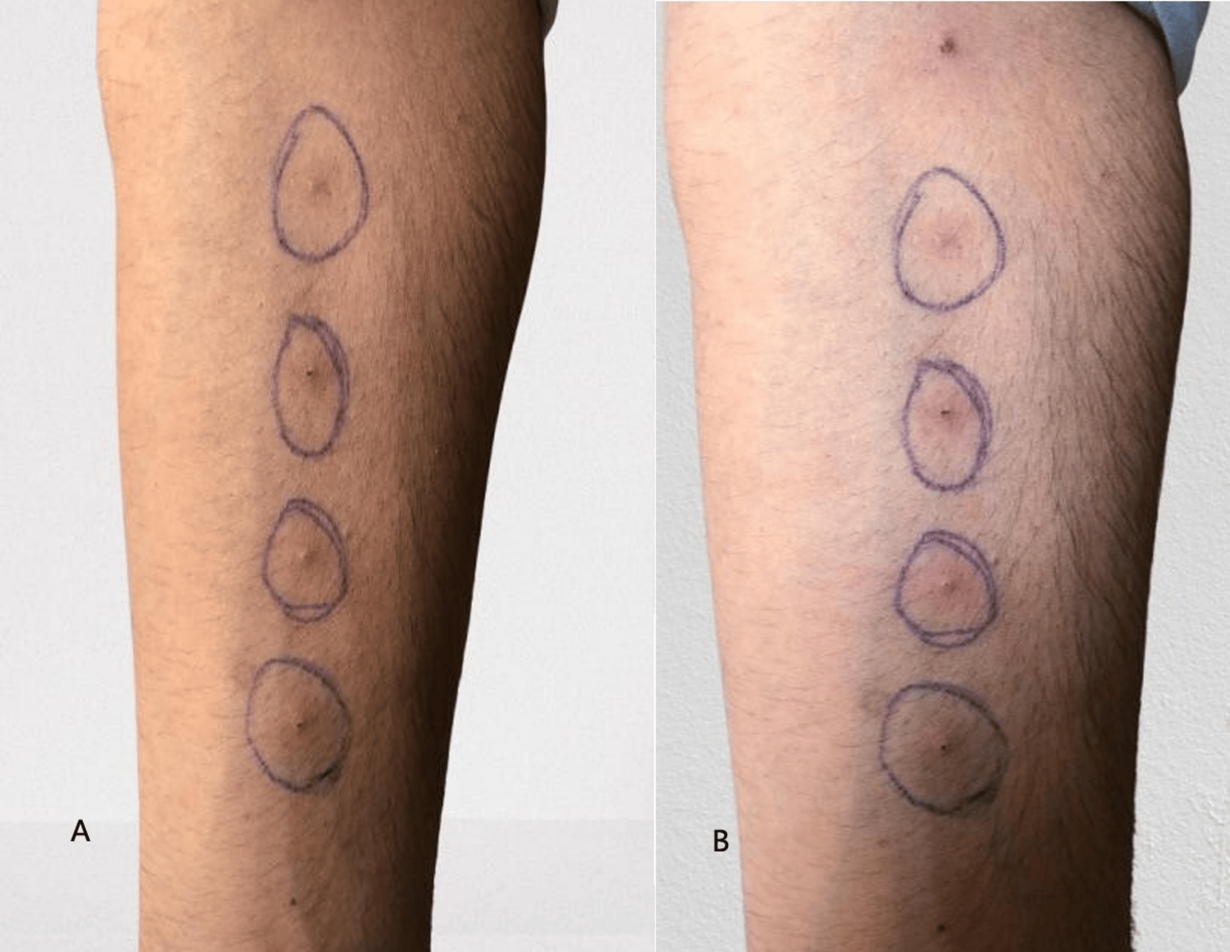
Pathergy test (A) Forearm showing four intradermal prick sites with pen markings; erythematous papules or pustules at 24 hours indicate a positive pathergy test. (B) Similar findings observed at 48 hours confirm a positive pathergy reaction.

A pathergy test was performed to support the diagnosis (Figure 2A, 2B). The test showed multiple erythematous papular lesions (2-3 mm in diameter) with surrounding erythema at the needle puncture sites within 48 hours, corresponding to a 1+ to 2+ positive reaction as per standard grading criteria (Table 3). This positive pathergy response is diagnostically significant, reflecting the abnormal hyperreactive inflammatory response characteristic of Behçet’s disease and fulfilling one of the minor diagnostic criteria of the International Study Group (ISG) classification.

**Table 3:** Characteristics of the pathergy reaction at 48 hours

Grades of SPT (SKIN PATHERGY TEST)	Blunt Needles	Sharp Needles
Negative (-)	Only Erythema or a trace of brick	Only Erythema or a trace of brick
Suspect (+)	Papule <2mm + erythema	Papule <3mm + erythema
Positive (+)		
1+	Papule 2-3mm + erythema	Papule <3mm + erythema
2+	Papule >3mm + erythema	Papule >3mm + erythema
3+	Pustule 1-2mm	Pustule 1-2mm
4+	Pustule >2mm	Pustule >2mm

The patient was managed with an intensive regimen of topical and systemic therapy. Initial treatment included topical corticosteroids in the form of prednisolone acetate eye drops administered hourly, along with cyclopentolate eye drops twice daily and topical nonsteroidal anti-inflammatory drugs (NSAIDs) twice daily. Systemic corticosteroid therapy was initiated with oral prednisolone at a dose of 0.5 mg/kg/day; however, this was subsequently discontinued following the development of corticosteroid hypersensitivity. Posterior subtenon injections of triamcinolone acetonide (40 mg/mL) were administered bilaterally during the initial phase of management.

Prior to the initiation of biologic therapy, pneumococcal and influenza vaccinations were administered, and screening for hepatitis B, hepatitis C, and tuberculosis (TB spot test) was completed, along with a baseline chest X-ray to exclude latent or active pulmonary infection (Table 4). The patient was then commenced on infliximab (5 mg/kg intravenously at 0, two, and six weeks, followed by maintenance infusions every six to eight weeks), a TNF-α inhibitor with established efficacy in the management of Behçet’s-related retinal vasculitis and panuveitis. Adalimumab (40 mg subcutaneously every two weeks) was identified as a potential alternative in the event of inadequate response or intolerance to infliximab.

**Table 4:** Vaccination and serologic screening prior to initiation of biologic therapy HBsAg – Hepatitis B surface antigen; ELISA – Enzyme-linked immunosorbent assay; IGRA – Interferon-gamma release assay.

Test / Vaccine	Date Administered / Performed	Product / Method	Result / Status
Pneumococcal vaccine	May 2025	PCV13 (Prevenar 13®)	Completed
Influenza vaccine	May 2025	Quadrivalent inactivated influenza vaccine (Vaxigrip Tetra®)	Completed
Hepatitis B surface antigen (HBsAg)	May 2025	ELISA	Negative
Hepatitis C antibody	May 2025	ELISA	Negative
Tuberculosis (TB) spot test	May 2025	Interferon-gamma release assay (IGRA)	Negative

**Figure 3:**
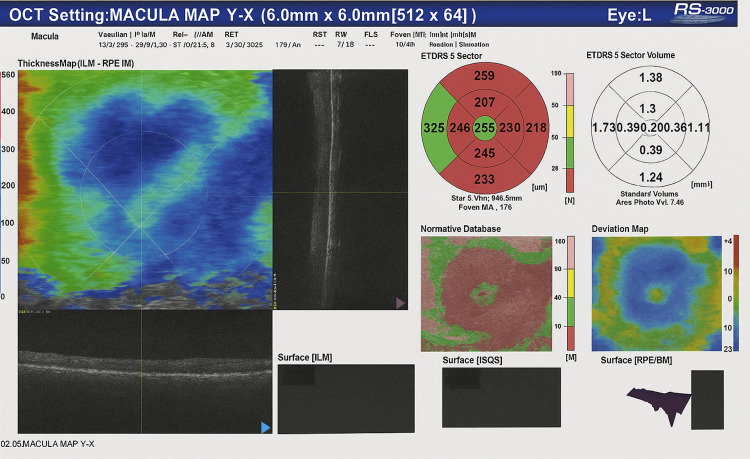
Optical coherence tomography (OCT), macula map of the left eye (L) showing retinal thickness distribution.

At the six-week follow-up after initiation of therapy, ocular examination demonstrated persistent bilateral intraocular inflammation consistent with uveitis. Visual acuity remained markedly reduced (counting fingers at 3 m in the right eye and 1 m in the left eye) with no improvement on pinhole testing. Active inflammatory signs were present, including a faint anterior chamber flare and Grade II vitreous haze. Chronic complications were also observed, such as bilateral posterior subcapsular cataracts (PSCC+) and posterior synechiae (pupillary adhesions to the lens) in the left eye (Table 5). Retinal involvement was evident, characterized by periphlebitis and vascular sheathing, findings suggestive of retinal vasculitis (Figure 3). Collectively, these features reflected a severe, panocular inflammatory process necessitating urgent and intensive immunosuppressive therapy.

**Table 5:** Comprehensive follow-up ocular examination, after six weeks, including both anterior and posterior segments (fundoscopic) findings. CF – Counting Fingers; PH – Pinhole; N/I – No Improvement; PSCC – Posterior Subcapsular Cataract; EOM – Extraocular Movements.

Visual Acuity	CF (3m)	CF (1m)
PH	N/I	N/I
EOM	Full	Full
Lids	Normal	Normal
Conjunctiva	Normal	Normal
Anterior Chamber	Faint flare	Faint flare
Cornea	Pigmented KPs	Endothelial dusting
Pupil	Pigment deposits on the lens	PS at 7,8,9,11,12
Lens	PSCC+	PSCC+
Vitreous	Vitreous haze grade II	Vitreous haze grade II
Retina	Peri-phlebitis	Peri-phlebitis
	Sheathing of vessels	Sheathing of vessels

Follow-up OCT showed marked anatomical improvement with restoration of the normal foveal contour and complete resolution of subretinal fluid. The central foveal thickness decreased to 198 µm (within normal range), and the total macular volume reduced to 7.16 mm³, indicating significant resolution of inflammatory changes and macular edema. These findings indicate substantial structural recovery of the macula and successful resolution of the serous detachment following therapy.

## Discussion

Behçet’s disease is a chronic, multisystem inflammatory disorder that is more prevalent in men than in women [8]. The disease typically begins with a constellation of overlapping symptoms. Approximately 20% of patients present initially with uveitis, and in about 40% of cases, it develops within two to three years of the onset of initial symptoms [9]. Ocular manifestations occur in approximately 30-70% of patients [10] with Behçet's disease, and contribute significantly to morbidity. Blindness develops in roughly 16-25% of such patients within five to 10 years of the onset of ocular symptoms [11].

Ocular involvement is characterized by recurrent, severe inflammatory attacks that tend to resolve spontaneously, leaving minimal or no signs of inflammation between episodes. In our case, the patient exhibited bilateral panuveitis, vitreous haze, posterior synechiae, and lens changes consistent with posterior subcapsular cataracts, resulting in visual impairment. These findings align with the characteristic ocular features of Behçet’s disease in male patients. The patient also reported recurrent oral and genital ulcers, the second most common manifestation, occurring in 57-93% of affected individuals [8].

Due to overlapping clinical features, Behçet’s disease is frequently misdiagnosed. A previous review on pseudo-Behçet’s disease reported that several conditions, such as complex aphthous dermatitis, herpes simplex virus infections, inflammatory bowel disease, Reiter’s syndrome, and tuberculosis, mimic Behçet’s disease [12].

Common clinical features shared by both tuberculosis and Behçet’s disease include fever, ulceration, weight loss, and central nervous system involvement, particularly in endemic regions such as Pakistan. Rarely, Behçet’s disease without active tuberculosis may present with caseating granulomas, histologically mimicking tuberculosis [13].

Currently, there is no single definitive diagnostic test for Behçet’s disease. However, diagnosis can be supported by a positive pathergy test, defined as the development of an erythematous papule (≥2 mm) or a pustule within 24-48 hours of minor trauma, reflecting exaggerated innate immune reactivity [14]. Diagnosis primarily relies on clinical evaluation. The ISG criteria, the most widely used since 1990, require recurrent oral ulcers plus any two of the following: genital ulceration, ocular lesions, skin lesions, or a positive pathergy test, demonstrating 95% sensitivity and 98% specificity. Later, the International Criteria for Behçet’s Disease (ICBD), introduced in 2013, employed a scoring system in which ocular, genital, and oral aphthous lesions are each assigned two points, while neurological and vascular manifestations, along with a positive pathergy test, receive one point each. A total score of 4 or greater confirms the diagnosis [15,16].

Our patient presented with oral aphthous ulcers, genital aphthous ulcers, uveitis, and a positive pathergy test, yielding a total score of 7 points according to the ICBD criteria (Table 6) [16].

**Table 6:** Summary of clinical features considered in the 2013 International Criteria for Behçet’s Disease (ICBD)

Clinical Domain	Examples of Findings	Relative Diagnostic Weight
Oral Mucosal Lesions	Recurrent aphthous ulcers	High
Genital Lesions	Recurrent genital ulcers, scarring	High
Ocular Involvement	Anterior/posterior uveitis, retinal vasculitis	High
Cutaneous Manifestations	Erythema nodosum–like lesions, papulopustular lesions	Moderate
Neurological Features	Parenchymal CNS disease, meningoencephalitis	Moderate–High
Vascular Manifestations	Arterial thrombosis, venous thrombosis, large-vessel disease	Moderate
Other Supportive Features	Pathergy reaction (if performed)	Optional
Classification Threshold	Combination of features reaching diagnostic weight	≥ 4 points required

To evaluate systemic involvement and confirm an autoimmune etiology, investigations including B-scan ultrasonography, anti-neutrophil cytoplasmic antibody (ANCA), cytoplasmic ANCA (C-ANCA), ESR, and chest radiography were performed to establish a comprehensive differential diagnosis. These were essential to distinguish Behçet’s disease from conditions with overlapping features, such as sarcoidosis, tuberculosis, and other autoimmune uveitic syndromes [9]. Tubercular uveitis was specifically excluded due to the patient’s prior history of antitubercular therapy and ocular findings of posterior synechiae and vitreous haze. Chest radiography, ESR, and ANCA testing were also vital to rule out vasculitic and granulomatous disorders.

The management of Behçet’s disease varies according to disease severity [10], duration, age, sex, and clinical manifestations [17]. Although systemic corticosteroids are considered the mainstay of therapy [8], their use was contraindicated in this patient due to a documented steroid hypersensitivity reaction characterized by a maculopapular rash and periorbital swelling. Therefore, alternative immunosuppressive therapy - including prednisolone [8] - was initiated as first-line systemic management to control acute flare-ups, followed by cataract surgery once ocular inflammation was stabilized. The patient was also prescribed triamcinolone acetonide, a potent local corticosteroid, to reduce intraocular inflammation and preserve visual function.

## Conclusions

This case emphasizes the need to distinguish Behçet’s disease from tuberculosis, as delayed recognition can lead to disease progression and visual loss. Careful pre-biologic screening for tuberculosis and other infections is essential before initiating TNF-α inhibitors. Ophthalmic warning signs such as recurrent uveitis, retinal vasculitis, or rapid visual decline warrant urgent evaluation. Although no curative therapy exists, timely immunosuppressive treatment can prevent irreversible ocular damage. Follow-up optical coherence tomography at three to six months is planned to assess anatomical stability and guide ongoing management.
